# Canada Medical Journal

**Published:** 1864-07

**Authors:** 


CANADA MEDICAL JOURNAL.—We have received the first number of a new Medical Journal, published in Montreal. Edited by G. E, Fenwick, M. D. and F.W. Campbell, M, D., L. R. C. P. S. It is to be published monthly, and contains forty eight pages.

We heartily congratulate the profession of Canada upon the appearance of this Journal. This number contains original matter of great interest, and this enterprise should enlist the interest of the whole Canadian profession.

